# Supplementary material for: Correlation between acute cellular rejection detected with cryobiopsy and elevated dd-cfDNA in lung transplant recipients
Source: JHLT Open. 2025 Dec 5;11:100454. doi: 10.1016/j.jhlto.2025.100454 (PMC12800636; doi:10.1016/j.jhlto.2025.100454)
Supplement: Supplementary file 1 — Supplementary material [file mmc1.docx]

**Table S1**. Microbiological culture results by ACR diagnosis.

|  | No ACR  (n=197) | | ACR  (n=43) | Total  (n=240) | p-value |
| --- | --- | --- | --- | --- | --- |
| Any bacterial detection | 126 (64.0%) | | 34 (79.1%) | 160 (66.7%) | 0.057 |
| Staphylococcus aureus |  | |  |  | 0.715 |
| 10^3^/ml | 1 (0.5%) | | 0 (0.0%) | 1 (0.4%) |  |
| 10^4^/ml | 2 (1.0%) | | 0 (0.0%) | 2 (0.8%) |  |
| Enterococcus faecalis |  | |  |  | 0.414 |
| 10^3^/ml | 3 (1.5%) | | 0 (0.0%) | 3 (1.3%) |  |
| Streptococcus parasanguinis |  | |  |  | **0.027** |
| 10^3^/ml | 27 (13.8%) | | 10 (23.3%) | 37 (15.5%) |  |
| 10^4^/ml | 0 (0.0%) | | 1 (2.3%) | 1 (0.4%) |  |
| Pseudomonas aeruginosa |  | |  |  | 0.914 |
| 10^3^/ml | 7 (3.6%) | | 1 (2.3%) | 8 (3.3%) |  |
| 10^4^/ml | 4 (2.0%) | | 1 (2.3%) | 5 (2.1%) |  |
| Streptococcus salivarius | |  |  |  | 0.955 |
| 10^3^/ml | | 28 (14.3%) | 6 (14.0%) | 34 (14.2%) |  |
| Staphylococcus epidermidis | |  |  |  | 0.700 |
| 10^3^/ml | | 15 (7.7%) | 2 (4.7%) | 17 (7.1%) |  |
| 10^4^/ml | | 1 (0.5%) | 0 (0.0%) | 1 (0.4%) |  |
| Actinomyces odontolyticus | |  |  |  | 0.778 |
| 10^3^/ml | | 34 (17.3%) | 7 (16.3%) | 41 (17.2%) |  |
| 10^4^/ml | | 2 (1.0%) | 1 (2.3%) | 3 (1.3%) |  |
| Streptococcus mitis | |  |  |  | 0.481 |
| 10^3^/ml | | 13 (6.6%) | 5 (11.6%) | 18 (7.5%) |  |
| 10^4^/ml | | 1 (0.5%) | 0 (0.0%) | 1 (0.4%) |  |
| Roth dent | |  |  |  | 0.460 |
| 10^3^/ml | | 5 (2.6%) | 2 (4.7%) | 7 (2.9%) |  |
| Lact rhamno | |  |  |  | 0.486 |
| 10^3^/ml | | 2 (1.0%) | 1 (2.3%) | 3 (1.3%) |  |
| Actinomyces oris | |  |  |  | 0.460 |
| 10^3^/ml | | 5 (2.6%) | 2 (4.7%) | 7 (2.9%) |  |
| Actinomyces graevenitzii | |  |  |  | 0.178 |
| 10^3^/ml | | 8 (4.1%) | 0 (0.0%) | 8 (3.3%) |  |
| Steptococcus viridans group | |  |  |  | 0.183 |
| 10^3^/ml | | 18 (9.2%) | 7 (16.3%) | 25 (10.5%) |  |
| 10^4^/ml | | 1 (0.5%) | 1 (2.3%) | 2 (0.8%) |  |
| Strp sanguinis | |  |  |  | 0.506 |
| 10^3^/ml | | 2 (1.0%) | 0 (0.0%) | 2 (0.8%) |  |
| Roth muclia | |  |  |  | 0.206 |
| 10^3^/ml | | 30 (15.3%) | 10 (23.3%) | 40 (16.7%) |  |
| Cirtob freu 1 | |  |  |  | 0.506 |
| 10^3^/ml | | 2 (1.0%) | 0 (0.0%) | 2 (0.8%) |  |
| Cirtob freu 2 | |  |  |  | 0.639 |
| 10^3^/ml | | 1 (0.5%) | 0 (0.0%) | 1 (0.4%) |  |
| Stph hominis | |  |  |  | 0.506 |
| 10^3^/ml | | 2 (1.0%) | 0 (0.0%) | 2 (0.8%) |  |
| Stph Haemol | |  |  |  | 0.713 |
| 10^3^/ml | | 3 (1.5%) | 1 (2.3%) | 4 (1.7%) |  |
| Strp gordonii | |  |  |  | 0.506 |
| 10^3^/ml | | 2 (1.0%) | 0 (0.0%) | 2 (0.8%) |  |
| Lactobac sp. | |  |  |  | 0.208 |
| 10^3^/ml | | 7 (3.6%) | 0 (0.0%) | 7 (2.9%) |  |
| Enterokokk faecium | |  |  |  | 0.509 |
| 10^3^/ml | | 5 (2.6%) | 0 (0.0%) | 5 (2.1%) |  |
| 10^5^/ml | | 1 (0.5%) | 0 (0.0%) | 1 (0.4%) |  |
| Bacillus sp | |  |  |  | 0.639 |
| 10^3^/ml | | 1 (0.5%) | 0 (0.0%) | 1 (0.4%) |  |
| Lactobac casei | |  |  |  | 0.639 |
| 10^3^/ml | | 1 (0.5%) | 0 (0.0%) | 1 (0.4%) |  |
| Act schaalii | |  |  |  | 0.639 |
| 10^3^/ml | | 1 (0.5%) | 0 (0.0%) | 1 (0.4%) |  |
| Klebsiella oxytoca | |  |  |  | 0.639 |
| 10^3^/ml | | 1 (0.5%) | 0 (0.0%) | 1 (0.4%) |  |
| Stenotrophomonas maltophilia | |  |  |  | 0.506 |
| 10^3^/ml | | 2 (1.0%) | 0 (0.0%) | 2 (0.8%) |  |
| Haemophilus parainfluenza | |  |  |  | 0.195 |
| 10^3^/ml | | 3 (1.5%) | 2 (4.7%) | 5 (2.1%) |  |
| Micrococcus luteus | |  |  |  | 0.506 |
| 10^3^/ml | | 2 (1.0%) | 0 (0.0%) | 2 (0.8%) |  |
| Staph warneri | |  |  |  | 0.639 |
| 10^3^/ml | | 1 (0.5%) | 0 (0.0%) | 1 (0.4%) |  |
| E. Coli | |  |  |  | 0.802 |
| 10^3^/ml | | 1 (0.5%) | 0 (0.0%) | 1 (0.4%) |  |
| 10^5^/ml | | 1 (0.5%) | 0 (0.0%) | 1 (0.4%) |  |
| Granulicat adiacens | |  |  |  | 0.486 |
| 10^3^/ml | | 2 (1.0%) | 1 (2.3%) | 3 (1.3%) |  |
| Apathogene Neisserien | |  |  |  | 0.932 |
| 10^3^/ml | | 5 (2.6%) | 1 (2.3%) | 6 (2.5%) |  |
| Strp pseudopneumoniae | |  |  |  | 0.639 |
| 10^3^/ml | | 1 (0.5%) | 0 (0.0%) | 1 (0.4%) |  |
| Act naeslundii | |  |  |  | 0.639 |
| 10^3^/ml | | 1 (0.5%) | 0 (0.0%) | 1 (0.4%) |  |
| Strp mutans | |  |  |  | 0.639 |
| 10^3^/ml | | 1 (0.5%) | 0 (0.0%) | 1 (0.4%) |  |
| Klebsiella pneumoniae | |  |  |  | 0.639 |
| 10^3^/ml | | 1 (0.5%) | 0 (0.0%) | 1 (0.4%) |  |
| Rothia sp. | |  |  |  | 0.639 |
| 10^3^/ml | | 1 (0.5%) | 0 (0.0%) | 1 (0.4%) |  |
| Strp anginosus | |  |  |  | 0.639 |
| 10^3^/ml | | 1 (0.5%) | 0 (0.0%) | 1 (0.4%) |  |
| Lact gasseri | |  |  |  | 0.237 |
| 10^3^/ml | | 1 (0.5%) | 1 (2.3%) | 2 (0.8%) |  |
| Enterococc raffinosus | |  |  |  | 0.639 |
| 10^4^/ml | | 1 (0.5%) | 0 (0.0%) | 1 (0.4%) |  |
| Dermabact hominis | |  |  |  | 0.639 |
| 10^5^/ml | | 1 (0.5%) | 0 (0.0%) | 1 (0.4%) |  |
| Corynebact accolens | |  |  |  | 0.639 |
| 10^5^/ml | | 1 (0.5%) | 0 (0.0%) | 1 (0.4%) |  |
| Bifidobact scardovii | |  |  |  | 0.639 |
| 10^3^/ml | | 1 (0.5%) | 0 (0.0%) | 1 (0.4%) |  |

Note: Total detection rates may not add up due to multiple infections within a patient

**Table S2.** Fungal cultural results by ACR diagnosis.

|  | No ACR  (n=197) | ACR  (n=43) | Total  (n=240) | p-value |
| --- | --- | --- | --- | --- |
| Any fungal detection | 38 (27.0%) | 15 (41.7%) | 53 (29.9%) | 0.085 |
| Aspergillus antigen | 0.08  0.06-0.10] | 0.07  [0.07-0.10] | 0.08  [0.07-0.10] | 0.512 |
| Candida glabrate | 3 (2.0%) | 1 (2.6%) | 4 (2.2%) |  |
| Aspergillus fumigatus | 2 (1.4%) | 3 (7.9%) | 5 (2.7%) |  |
| Penicillium sp | 12 (8.2%) | 5 (13.2%) | 17 (9.2%) |  |
| Aspergillus nidulans | 1 (0.7%) | 0 (0.0%) | 1 (0.5%) |  |
| Candida albicans | 3 (2.0%) | 2 (5.3%) | 5 (2.7%) |  |
| Penicillium sp & Aspergillus glaucus | 1 (0.7%) | 0 (0.0%) | 1 (0.5%) |  |
| Penicillium sp & Aspregillus ochraceus | 1 (0.7%) | 0 (0.0%) | 1 (0.5%) |  |
| Aspergillus ochraceus & Aspergillus versicolor | 0 (0.0%) | 1 (2.6%) | 1 (0.5%) |  |
| Cladosporium sp | 1 (0.7%) | 0 (0.0%) | 1 (0.5%) |  |
| Candida albicans, Trichoderma sp & Penicillium sp | 1 (0.7%) | 0 (0.0%) | 1 (0.5%) |  |
| Aspergillus niger & Penicillium sp | 1 (0.7%) | 0 (0.0%) | 1 (0.5%) |  |
| Candida albicans, Candida glabrata & Hypocreales | 1 (0.7%) | 0 (0.0%) | 1 (0.5%) |  |

Note: Total detection rates may not add up due to multiple infections within a patient.

**Table S3.** Viral detection rates by ACR diagnosis.

|  | No ACR | ACR | Total | p-value |
| --- | --- | --- | --- | --- |
| Any viral detection | 24 (12.8%) | 7 (16.7%) | 31 (13.5%) | 0.512 |
| Parainfluenza 4 | 2 (1.1%) | 0 (0.0%) | 2 (0.9%) | 0.501 |
| CMV | 0 (0.0%) | 1 (2.4%) | 1 (0.4%) | **0.033** |
| EBV DNA | 1 (0.5%) | 0 (0.0%) | 1 (0.4%) | 0.638 |
| Human Rhinovirus/Enterovirus | 13 (6.7%) | 3 (7.1%) | 16 (6.8%) | 0.924 |
| SARS-COV2 | 3 (1.5%) | 0 (0.0%) | 3 (1.3%) | 0.413 |
| Coronavirus NL63 | 2 (1.0%) | 1 (2.4%) | 3 (1.3%) | 0.482 |
| Coronavirus HKU1 | 1 (0.5%) | 1 (2.3%) | 2 (0.8%) | 0.240 |
| Coronavirus 229E | 0 (0.0%) | 1 (2.3%) | 1 (0.4%) | **0.033** |
| Parainfluenzae 3 | 1 (0.5%) | 0 (0.0%) | 1 (0.4%) | 0.638 |
| Influenza A | 1 (0.5%) | 0 (0.0%) | 1 (0.4%) | 0.638 |

Note: Total detection rates may not add up due to multiple infections within a patient.
